# Monthly Summary

**Published:** 1892-03

**Authors:** 


### Monthly Summary.

ELECTRICITY IN TOOTH EXTRACTION.—A small party of medical men and dentists lately met at the Institute of Medical Electricity, 35 Fitzroy Square, W. C., London, to witness a demonstration of the new method of extracting teeth without pain. One of our staff was there. We sent the one who has most experience in the shocks and squirms of the dentist's chair, and he was imbued when he left the office with more than his share of skepticism regarding the powers of electricity in drawing teeth. He came back brimming full of enthusiasm about the "vibrator." That is what the electrical arrangement is called. It is a simple arrangement, consisting of a neat walnut case, within which are a couple of bichromate cells and a Ruhmkorff coil to which is attached a commutator of extreme sensitiveness. The commutator is the secret of the whole affair. It is a thin ribbon of highly tempered metal, secured at each end by an elaborate arrangement of screws. It is capable of vibrating at a tremendous pace—so quickly, indeed, that it is really musical—and the force passing through the coil is regulated until the vibrator is in unison with the key A, which the Philharmonic Society says is equal to 420 vibrations per second.

The operator was Mr. Burgoyne Pillin, L. D. S., who stated that he was a visitor himself, not being connected with the institute. He has four patients in waiting. The first was a young professional man, who seated himself in the operating chair to get a bicuspid extracted. He got the handles of the battery in his hands. One of these is connected with the negative pole. The positive is divided into two, so that one of the divisions is connected with the handle and a wire from the other division is screwed into the handle of the tooth forceps. When the patient takes hold of the handles the current is gradually increased in intensity until the patient can bear no more; then, while the forceps are being introduced, the current is turned off for a second and on again. The rest is the same as without electricity. "Had you no pain?" asked our representative of the patient when the roots of the bicuspid were held up to view. "Not a bit; I only felt the grip." "What did you mean by stretching your body, then?" "Oh, that was when the current was turned on." "You didn't feel the frightful wrench, then?" "No," was the reply. Our representative was still skeptical, it will be seen. All this skepticism went with the next patient, a young and robust-looking lady, who had the left anterior upper molar troubling her. She took the chair, and quickly enough Mr. Pillin had his forceps on the shell. Crack it went, and the usual thing followed—three separate extractions, the last bringing away part of the crown and two twisted roots an inch in length—as bad a case as one could wish to see. It took some time to persuade the patient that her tooth was out. "I felt no pain," she said, after she had an affirmative reply to her question, "Is it out?" The next patient was a young lad who declared that he felt like getting a shave (he had not got his first). His lower bicuspid was also quickly brought to view and he went out with a smile.

The next turned out to be a bad case. The tooth was fearfully exostosed, and it was only by a prolonged wrench, which was painful to look upon, that Mr. Pillin got it out; but the patient showed not a trace of pain, and he, like the others before him, was quite free from shock. This is one of the characteristics of the process: there is no nervous shock.

The four cases were typical, and all the experts present were enthusiastic about the success, and loud in their praises of Mr. Pillin's skill. Now, why is it that electricity prevents pain? was the question that every one was asking. "Simple enough," said Dr. Arthur Harries, the physician in the institute. "Electricity travels over the nerve at the rate of 420 vibrations per second; pain travels from the tooth to the brain in one-sixtieth of a second. My theory is that the electricity, being so much quicker and having the greatest force behind it, gets to the brain first, and then keeps the line for itself, crowding out the pain." If Dr. Harries' theory is right, what a future there is for electricity in surgery! Chloroform and all other anæsthetics will have to take a back seat, and we shall banish pain simply by not allowing it to be produced.

There are other points about the vibrator which we should like to speak of, but need only mention that there is less bleeding and that it interferes in no way with the operator. It is really a good thing, thoroughly sound in principle and without any humbug about it.—*Chemist and Druggist.*

---

TWO COLLATERAL CASES.—By J. D. Ross Watt, L. R. C. S., L. R. C. P., L. D. S.—I have to bring before you the facts of two cases, which occurred under my notice lately, of irritation produced by the presence of a foreign body, the first case occurring in a patient, in the second instance, curiously enough the subject is a calf. It will be observed how analogous are the two cases, as they present themselves.

CASE I.

Mr.—— presented himself complaining of pain attendant upon and referred to the left lower wisdom tooth. The teeth are examined but nothing is visible to explain the symptoms present which are pain, tenderness, and slight swelling and redness, though thought to be of a neurotic nature. Next day patient again seeks another consultation, and on examination for a second time, the gum on the posterior surface of the wisdom is noticed to be inflamed, tender to pressure and slightly indurated. On further inspection there is found between the tooth itself and the gum, a bristle of a tooth brush which has insinuated itself here. This is withdrawn, relief is obtained and symptoms subside and matters are explained. Showing us how careful should we be in our methods of examinations. The time which elapsed from the commencement of the pain, felt to the time when the bristle is removed is one week.

CASE II.

Side by side with the above I place the following. Mr. ——, butcher, has a calf which he takes before the veterinary surgeon, seeking explanation about a peculiar swelling situated about the region of the mouth and jaws, in consequence of which the animal though apparently in good health refuses to partake of food. The matter is unexplainable. The butcher having the above history is rather chary of selling the head of the animal (which are used to make a preparation known as potted head). However on the head being cut up and dissected, attention is turned to the jaw and mouth and there is found on the buccal side of a right upper tooth, a piece of straw which measures, on being withdrawn, 1½ inches in length.

There is also a considerable amount of swelling and induration present, and a slight amount of pus can be made to exude on pressure being applied to the gum round the tooth. From the above history the discomfort of the animal can easily be accounted for.—*Dent. Record.*

---

EUCALYPTO RESORCIN.—This is the name given by Barbey, in the *Bulletin Commercial*, to a reaction product of resorcin and oil of eucalyptus. Resorcin in excess is mixed with oil of eucalyptus, and chloroform is added, and the whole shaken. The clear solution, when poured off, leaves behind a mass of needle-like crystals, having a strong camphoric odor, and which are insoluble in water, but easily soluble in alcohol, ether and chloroform. This body, on fractional distillation, gives various liquid and solid distillates which have not yet been examined. Naphthol, pyrogallol, and picric acid also give with eucalyptol similar crystalline bodies, all of which have a strong camphoric odor.—*Med. and Surg. Reporter.*

---

A STUDENT of pharmacy in Hesse, was called upon to put up a prescription containing a 20 per cent. solution of chromic acid, salicylic acid and water. He put the chromic acid directly into the water and is now nursing numerous burns, the result of the explosion which followed.

THE IMPLANTATION OF ARTIFICIAL TEETH.—Dr. A. H. Rainey, Centralia, Ill., was in the city recently, and we asked him how the teeth with metallic roots were doing. He replied that they were still giving satisfaction. The doctor read a paper on the subject last October at the Southern Illinois Dental Society. At that time, he stated that the oldest case he had was inserted eighteen months previous, and was doing good service. He uses block tin, cast on a Logan crown. The metallic root is enlarged at a point one-third of an inch from the neck of the tooth, making it of such a shape as to be retained in the alveolus, mechanically; the root is polished before inserting.

The *Deutsche Monatsschrift fur Zahnheilkunde*, March, 1891, contains an interesting, but lengthy article on "The Implantations of Artificial Teeth," by Dr. N. N. Znamensky. They were tested by Professor Sklifosoffsky and A. B. Vogt, neither of whom could with two fingers shake or remove the tooth. In a third case, the dog gnawed at its chain before the tooth was firmly fixed, and the ultimate result was not so good. Dr. Znamensky has only tried this in one instance on a human subject, and that was not a favorable case, as the anterior alveolar wall was absent, and the tooth had already been extracted some six weeks, and consequently the socket had to be drilled out. A month later the tooth was, however, fairly firm, but as the patient had to leave Moscow, he was not seen again. He was to write, if any unfavorable symptoms arose, but not having done so, Znamensky concludes they have not occurred."—*Archives of Dentistry.*

---

DEATH FROM COCAINE INJECTION.—The *Journal fur Zahnheilkunde* reports a case of death in a dentist's chair from injection of cocaine into the gum, given for the purpose of inducing anæsthesia for the extraction of roots of teeth. The patient was a woman, 29 years old, apparently perfectly healthy, but very nervous. The extraction was painless, and nothing abnormal was noted. The operator withdrew from the patient's chair to get some water for the patient to rinse her mouth with, and on his return found her motionless. Physicians were summoned and artificial respirations was practiced, but without success. The autopsy disclosed the fact that three injections had been given, which served for the extraction of three roots. The quantity of cocaine in each injection was two centigrams, or one-third of a grain. The *Journal*, after commenting upon the dangers of cocaine, refers to nine cases of fatal poisoning reported by Dufournier in the *Archives generales de Medicine*. One of these cases, however, is doubtful, as the patient took a mixture of chloral and cocaine. The *Medical and Surgical Reporter*, in quoting the above, says that the uncertainty of the action of cocaine is shown in a case reported in the *British Medical Journal*, where one-seventh of a grain injected into the eyelid produced very serious poisoning.—*D. & S. Microcosm*.

---

PLASTER OF PARIS FORMULA.—1. *To Make Plaster Set Hard*.—Mix best plaster of Paris with about 10 per cent. (more or less, according to effect ascertained by preliminary experiment) of very finely powdered marble (calcium carbonate). Or add to it about 6 per cent. of powdered alum, or about the same amount of ammonium chloride, before mixing with water.

2. *To Make Plaster Set Slower*.—Mix it with 2 to 4 per cent. of powdered althæa root before adding the water, This not only retards the hardening of the plaster, but also enables it to be cut, filed, sawed and turned.

An addition of 8 per cent. of althæa powder retards the complete setting of the plaster for about one hour, so that the mass can be used for any purpose where it is to remain plastic during at least a portion of that time.—*Amer. Drug*.

